# Assessment of internal consistency reliability and concurrent validity of PROMIS^®^ Dyspnea Characteristics v1.0, PROMIS^®^ Dyspnea Functional Limitations 10a, and PROMIS^®^ Global Health v1.2 in a dysautonomia cohort

**DOI:** 10.1186/s41687-026-01134-w

**Published:** 2026-07-08

**Authors:** Andrew J. Latham, John A. O’Dell, Thanh Ho, Shelley Chavis, Silvia E. Smith

**Affiliations:** University of North Carolina at Pembroke, Pembroke, USA

**Keywords:** Dyspnea, Dysautonomia, Patient-reported outcomes, Instrument validation

## Abstract

**Background:**

Dyspnea, or shortness of breath, is frequently reported among individuals with autonomic nervous system dysfunction, or dysautonomia. Currently, there are no validated dyspnea-specific patient-reported outcome measures for this population.

**Methodology:**

We conducted a patient-reported outcomes study to evaluate the internal consistency, reliability, and concurrent criterion-related validity of three PROMIS^®^ instruments: Dyspnea Characteristics v1.0, Dyspnea Functional Limitations 10a, and Global Health v1.2 in a cohort of adult individuals with dysautonomia. These instruments assess the prevalence of dyspnea, general quality of life, and dyspnea-related functional limitations.

**Results:**

Of the 617 participants enrolled in this study, 414 individuals without comorbid conditions causing dyspnea were included in the primary analysis. The PROMIS^®^ Dyspnea Characteristics v1.0 and PROMIS^®^ Dyspnea Functional Limitations 10a instruments demonstrated good to excellent internal consistency reliability (Cronbach’s alpha = 0.896-0.909; McDonald’s omega total = 0.911-0.912). The PROMIS^®^ Global Health v1.2 instrument demonstrated good overall internal consistency (Cronbach’s alpha = 0.846, McDonald’s omega total = 0.852), although reliability varied among the Global Physical Health and Global Mental Health summary scores (Cronbach’s alpha = 0.640-0.759; McDonald’s omega total = 0.664-0.767). Confirmatory factor analysis supported a unidimensional structure for both the PROMIS^®^ Dyspnea Characteristics v1.0 and Dyspnea Functional Limitations 10a instruments and a two-factor structure (Global Physical Health and Global Mental Health) for the Global Health v1.2 instrument. The indices demonstrated a good to excellent model fit (Comparative Fit Index = 0.983-1.00). Analyses demonstrated strong concurrent criterion-related validity for the PROMIS^®^ Dyspnea Functional Limitations 10a instrument (r = 0.59063) and weak-to-moderate concurrent validity for the Global Health v1.2 instrument (r = -0.17763 to -0.35034).

**Conclusion:**

These findings support the use of the PROMIS^®^ Dyspnea Functional Limitations 10a and Global Health v1.2 instruments in clinical and research settings to assess symptom burden, functional limitations, and quality of life in adults with dysautonomia.

## Background

### Prevalence of dyspnea in dysautonomia

Dysautonomia is an umbrella term that includes any condition hindering the anatomy and physiology of the autonomic nervous system (ANS), with postural orthostatic tachycardia syndrome (POTS) being the most commonly diagnosed type [1]. Patients with dysautonomia may experience a wide variety of symptoms that can substantially impair daily functioning and quality of life. These symptoms reflect the central and vital role of the ANS in cardiovascular, respiratory, and airway regulation.

Dyspnea, or shortness of breath, is frequently reported among individuals with ANS dysfunction. Research shows that its prevalence estimates can vary by type of autonomic disorder, sample size, and assessment methodology. For example, dyspnea has been reported in approximately 30% of patients with orthostatic hypotension in a cohort of 651 participants [2], 31% of patients with orthostatic intolerance in a cohort of 100 [3], and 37% and 64% of patients with POTS in cohorts of 64 and 87 participants, respectively [3, 4]. While these studies focused on data reported from homogeneous groups of dysautonomia patients, they illustrate that dyspnea is a rather common and clinically relevant symptom across multiple types of autonomic dysfunction.

### Pathophysiology of dyspnea in dysautonomia

Several pathophysiological processes may explain dyspnea in people with ANS dysfunction and associated orthostatic intolerance, or the inability to withstand an upright position. The main driver of dyspnea is central or thoracic hypovolemia, a physiological state characterized by low blood volume in the chest cavity. In people with ANS dysfunction, thoracic hypovolemia may result from an impaired renin-angiotensin-aldosterone enzyme system [5] and/or vascular denervation [6]. Dyspnea may result from inadequate ventricular filling (i.e., preload failure) and stroke volume associated with thoracic hypovolemia [7, 8].

**Fig. 1:**
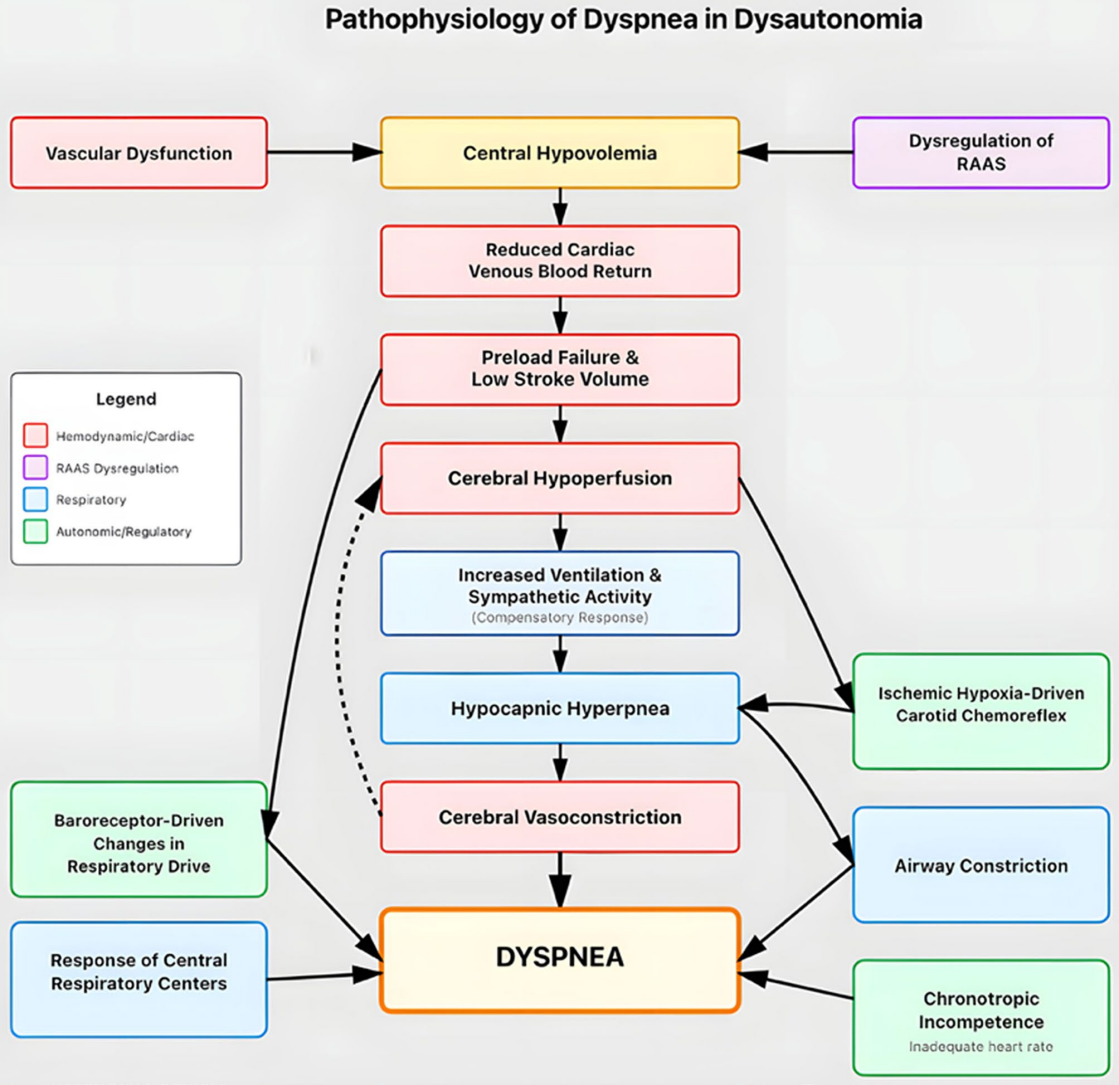
Pathophysiology of dyspnea in dysautonomia [15]

Dysautonomia and associated orthostatic intolerance are also characterized by cerebral hypoperfusion, or reduced blood flow velocity to the brain, which may also be associated with dyspnea. Cerebral hypoperfusion results from low stroke volume, which in turn leads to hypocapnia during orthostatic stress, either directly by increasing compensatory hyperventilation and sympathetic activity [8], or indirectly via intermittent ischemic hypoxia of the carotid body. This in turn activates chemoreflex pathways that increase breathing rate and depth (i.e., postural hypocapnic hyperpnea) [9–11]. In addition to causing dyspnea, postural hypocapnic hyperpnea can perpetuate cerebral hypoperfusion by causing cerebral vasoconstriction [9]. Other mechanisms leading to postural hypocapnia in dysautonomia patients have also been proposed, including orthostatic-ventilation perfusion mismatch, baroreceptor-driven changes in respiratory drive, chronotropic incompetence, and alteration in central respiratory center responsiveness [1, 12]. Finally, since airway caliber regulation is under the control of the ANS [13], it is conceivable that dysregulated airway contractility may be associated with hypocapnia or autonomic dysfunction [14]. These pathophysiological mechanisms may occur in isolation or in combination (Fig. 1).

### Current gap: lack of validated dyspnea instruments

Dysautonomia patients may experience dyspnea with mild exertion or even at rest [7, 16]. Persistent dyspnea of any etiology is potentially an isolating factor that can impact quality of life, as individuals who experience persistent dyspnea tend to avoid activities that trigger or aggravate the severity of this symptom [17]. While the burden of dyspnea in cardiopulmonary disease is widely studied, there are no systematic assessments of dyspnea in people with dysautonomia. This may reflect the absence of standardized and psychometrically validated dyspnea-specific instruments for this patient population.

### Study aims

To address this need, we aimed to validate three Patient-Reported Outcomes Measurement Information System (PROMIS^®^) survey instruments that can be employed in clinical and research settings to assess the burden of dyspnea and its impact on quality of life in people with dysautonomia: Dyspnea Characteristics v1.0 [18], Dyspnea Functional Limitations 10a, [18] and Global Health v1.2 [19].

## Methods

### Dysautonomia cohort

To validate the PROMIS^®^ Dyspnea Characteristics v1.0, Dyspnea Functional Limitations 10a, and Global Health v1.2 instruments, we enrolled 617 willing participants in collaboration with several nonprofit dysautonomia organizations over a period of six months (January to July 2023). Enrollment for this study was described more extensively elsewhere [20], and it was conducted in accordance with the principles outlined in the Belmont Report, [21]. Declaration of Helsinki [22], and 45 CFR 46 (Common Rule) [23] Briefly, to meet inclusion criteria, participants had to: (1) be 18 years of age or older, (2) have a formal diagnosis of any type of ANS dysfunction, (3) be able to read and write English, and (4) autonomously consent to participate in a research study. As this is a no greater than minimal risk study, participants who met the inclusion criteria listed in the screening questionnaire self-consented by reading and electronically signing the Informed Consent Form online. Data from all instruments were collected and managed using REDCap electronic data capture tools [24] and housed on secure servers.

### Instruments

The National Institutes of Health PROMIS^®^ program provides validated measures of patient-reported outcomes related to common health conditions that have been validated in the general population [25]. We employed three PROMIS^®^ instruments as well as a custom instrument. Additionally, we standardized scoring and ensured conceptual alignment across the three PROMIS^®^ instruments.

### PROMIS^®^ Dyspnea Characteristics v1.0

This is a five-item instrument that evaluates qualitative and quantitative measures of presence, intensity, frequency, duration, and severity of dyspnea. Items use a 0–10 numeric rating scale or a 0–4 Likert scale to assess dyspnea severity over the course of the last 7 days. This instrument was analyzed using a raw summed score derived from participant item-level responses, consistent with PROMIS^®^ scoring guidance for Dyspnea Characteristics v1.0 [18, 26].

### PROMIS^®^ Dyspnea Functional Limitations 10a

These ten Likert-based questions assess the impact dyspnea has on an adult’s ability to perform daily activities across light, moderate, and heavy activity levels. This instrument is scored using Item Response Theory (IRT) to produce a standardized T-score, where an average score is 50, one standard deviation (SD) is 10, and higher scores indicate worse health [18, 27]. If a study participant attempted a functional task, these questions were numerically coded for scoring and CFA analyses, while PROMIS^®^ IRT scoring was used to derive the standardized T-scores (Appendix A1.1).

### PROMIS^®^ Global Health v1.2

This instrument is a 10-item survey, and it is designed to evaluate overall health as well as health-related quality of life across multiple domains (e.g., physical function, fatigue, pain, emotional distress, and social health). All items have a 5-point Likert response scale. This instrument yields two four-item summary scores: Global Physical Health (GPH) and Global Mental Health (GMH). The GPH score is calculated from four items assessing physical health, physical function, pain, and fatigue; the GMH score is calculated from four items assessing quality of life, mental health, social satisfaction, and emotional problems. Higher T-scores indicate better perceived health and functioning. For the GPH and GMH domains, item responses are scored using IRT to generate standardized T-scores, where, in the general population, the mean is 50 (SD = 10) [19, 28, 29].

It should be noted that two additional items, Global01 (Global General Health) and Global09 (Global Social Health), were treated as descriptive single-item indicators. These items are retained as standalone components of the PROMIS^®^ Global Health v1.2 instrument but are not included in the derivation of either the GPH or GMH summary scores. Nevertheless, they provide ancillary information regarding participants’ overall perceived health and social well-being [19, 28, 29].

For this study, items were scored according to PROMIS^®^scoring guidelines [19]. Items with negatively worded response scales (e.g., higher values indicate poorer health) were reverse-coded prior to analysis to ensure consistent interpretation across indicators. Following reverse coding, higher scores uniformly reflected better health, which facilitated interpretation and supported the estimation of reliability metrics and confirmatory factor analysis (CFA).

### Dysautonomia and Comorbid Conditions instrument

This is a custom instrument that identifies relevant autonomic diagnoses/disorders and common comorbid conditions for inclusion and exclusion purposes, including those that are also associated with dyspnea (Appendix A2.1).

### Statistical analysis

All statistical analyses were performed using R Project for Statistical Computing (version 4.5.1) [30]. The *psych* [31] and *semTools* [32] packages were used for reliability and validity metrics, while the *lavaan* [33, 34] package was used for CFA. All tests performed were two-tailed, and statistical significance was defined at *p* < 0.05. Since the PROMIS^®^ instruments have been validated in the general population, but not specifically in people with dysautonomia, we evaluated both internal consistency reliability and construct validity in people affected by any type of autonomic disorders. The Dysautonomia and Comorbid Conditions Instrument allowed us to identify participants affected by confounding dyspnea-associated comorbid conditions (e.g., asthma, cardiopulmonary disease, and severe anemia), and we removed those participants from subsequent statistical validation analyses (Table 3). The PROMIS^®^ Dyspnea Characteristics v1.0 raw composite score served as the independent measure of dyspnea severity, which was used to examine associations with the responses from both PROMIS^®^ Global Health v1.2 and Dyspnea Functional Limitations 10a instruments.

### Internal consistency reliability

For both PROMIS^®^ Dyspnea Functional Limitations 10a and Global Health v1.2 instruments, we assessed internal consistency reliability for each multi-item summary score using Cronbach’s alpha (α) and McDonald’s omega total (ω_t_). Following current psychometric standards, interpretations of Cronbach’s alpha regarding internal consistency reliability were 0.70–0.79 (acceptable), 0.80–0.89 (good), and ≥ 0.90 (excellent) [35]. Item–total correlations and “alpha if item deleted” values were examined to ensure that each question contributed meaningfully to its respective scale. McDonald’s omega total values ≥ 0.70 were interpreted as acceptable evidence in determining internal consistency reliability.

### Construct-related validity

To assess construct-related validity, we performed CFA on the three PROMIS^®^ instruments. Based on prior literature on instrument validation in the general population, we modeled the PROMIS^®^ Dyspnea Characteristics v1.0 and PROMIS^®^ Dyspnea Functional Limitations 10a as unidimensional constructs [18] and Global Health v1.2 as a two-factor structure, representing physical and mental health [19].

Models were estimated using diagonally weighted least squares (DWLS), which is an appropriate method for ordinal data. Due to our large sample size, we did not rely on the chi-square statistic to evaluate model acceptability. Instead, model fit was assessed using four indices, following Hu and Bentler’s recommended cutoffs [36]. Comparative Fit Index (CFI) and Tucker–Lewis Index (TLI) with values ≥ 0.95 indicate good fit. Root Mean Square Error of Approximation (RMSEA) and Standardized Root Mean Square Residual (SRMR) with values ≤ 0.08 are considered acceptable [37]. Where indicated, theoretically justified residual correlations were introduced to improve model fit. These responses may reflect a missing not at random (MNAR) mechanism in the PROMIS^®^ Dyspnea Functional Limitations 10a instrument, as individuals who experience greater dyspnea severity are less likely to engage in physically demanding activities [23]. We estimated CFA models using pairwise deletion, such that all available data were used for each pairwise covariance. Factor loadings were evaluated to ensure that all items contributed significantly to their latent constructs.

### Concurrent criterion-related validity

Concurrent criterion-related validity was examined by correlating PROMIS^®^ Dyspnea Characteristics v1.0 raw composite scores (indicating dyspnea severity) with PROMIS^®^ Dyspnea Functional Limitations 10a T-scores and PROMIS^®^ Global Health v1.2 summary T-scores (GPH and GMH), as well as descriptive single-item domains (Global01 [Global General Health] and Global09 [Global Social Health]). Pearson’s correlation coefficients (r) were interpreted as follows: 0.10–0.29 = weak, 0.30–0.49 = moderate, and ≥ 0.50 = strong associations [37]. This approach provided complementary evidence of the degree to which perceived dyspnea severity is associated with physical and psychosocial functioning within this patient population.

### Missing scores

Missing data in the PROMIS^®^ Dyspnea Functional Limitations 10a instrument resulted from survey responses marked as “did not do this activity in the last 7 days,” and these responses were treated as missing in the primary analysis, consistent with PROMIS^®^ scoring guidelines. Since the reason for not performing the activity cannot be definitively determined, assigning a difficulty score could potentially introduce a misclassification bias. However, these responses might reflect an MNAR mechanism, as patients with greater dyspnea severity may be less likely to attempt certain physically demanding activities. The primary CFA used DWLS with pairwise deletion, which assumes that data are missing at random (MAR). Reliability analyses were conducted using available data with pairwise complete observations, consistent with the CFA estimation approach. To assess robustness to this assumption, we conducted a sensitivity analysis in which the “did not do” responses were recoded to the highest difficulty level (score = 5, which reflects an inability to perform an activity). The CFA was again estimated using the same model specification. The sensitivity analysis results demonstrated minimal changes in factor loadings, reliability estimates, as well as model fit indices (Appendix 3). These findings indicate that the results were robust to alternative assumptions regarding missing data.

## Results

### Cohort characteristics

The demographic structure and description of autonomic disorders and comorbid conditions of this cohort have been previously described [20]. Updated and expanded data are presented in Tables 1 and 2. Briefly, a total of 617 adult participants met the inclusion criteria and completed all survey instruments. Among these, 414 participants reported no comorbid conditions known to cause dyspnea and were therefore included in the primary analysis; the distribution of dysautonomia types in this cohort is shown in Table 2. This subgroup was used for the psychometric evaluation of the PROMIS^®^ instruments in individuals with dysautonomia. The remaining 203 individuals identified one or more of the dyspnea-associated comorbid conditions listed on the Dysautonomia and Comorbid Conditions instrument and were excluded from subsequent analyses (Table 3).

**Table 1:** Cohort demographics (*n* = 414)

Demographics	Frequency (*n*)	Prevalence (%)
Ethnicity		
Hispanic or Latino	25	6.0
Not Hispanic or Latino	376	90.8
Unknown / Not reported	13	3.1
Race		
American Indian / Alaskan Native	1	0.2
Asian	4	0.9
African	2	0.4
Caucasian / European	378	91.3
More than one race	16	3.8
Unknown / Not reported	13	3.1
Gender		
Male	20	4.8
Female	377	91.0
Non-binary	13	3.1
Not reported	4	0.9

**Table 2:** *Types of dysautonomia* (*n* = 414). Multiple conditions may apply to each participant

Autonomic disorder	Frequency (*n*)	Prevalence (%)
Postural orthostatic tachycardia syndrome	377	77.0
Orthostatic hypotension	72	17.3
Neurocardiogenic syncope	58	14.0
Inappropriate sinus tachycardia	50	12.0
I don’t know / diagnosis in progress	37	8.9
Other dysautonomia	23	5.5
Secondary to a rheumatological condition	29	7.0
Other immune-mediated dysautonomia	24	5.7
Pure autonomic failure	3	0.7
Familial dysautonomia	7	1.6
Autoimmune autonomic ganglionopathy	3	0.7
Baroreflex failure	3	0.7
Multiple system atrophy	2	0.4

**Table 3:** *Comorbid conditions associated with dyspnea* (*n* = 203). Multiple conditions may apply to each participant. Participants who reported one or more of these conditions were removed from subsequent statistical analyses

Comorbid Condition	Frequency (*n*)	Prevalence (%)
Asthma	175	67.8
Obesity	61	23.6
Severe anemia	34	13.2
Other	33	12.8
Neuromuscular disease	22	8.5
Chronic obstructive pulmonary disease	14	5.4
Pulmonary embolism	13	5.0
Obstructive sleep apnea	13	5.0
History of heart attack	13	5.0
Heart valvular disease	12	4.7
Heart failure	11	4.3
Coronary artery disease	11	4.3
Pulmonary hypertension	9	3.5
Cardiomyopathy	8	3.1
Other heart disease	8	3.1
Mitochondrial disease	7	2.7
Interstitial lung disease	5	1.9
Other lung disease	5	1.9
None apply	1	0.4
Lung cancer	0	0.0

### Item analysis and internal consistency reliability

To evaluate the psychometric performance of the PROMIS^®^ instruments in our dysautonomia cohort, we conducted a series of item-level reliability analyses across the three PROMIS^®^ instruments.

### PROMIS^®^ Dyspnea Characteristics v1.0

We examined the contribution of each item to the overall scale reliability. At the item level, “alpha if item deleted” analyses confirmed that no item improved the reliability of the overall scale if removed. The corrected item-total correlations ranged from 0.83 to 0.89, which indicates that each item demonstrates a strong positive association with the total score when the item itself is excluded. Moreover, the “Cronbach’s alpha if item deleted” values were lower than the overall scale Cronbach’s alpha. This further supports the homogeneity of item responses. Both values (α = 0.896; ω_t_ = 0.911) indicate good to excellent internal consistency within the PROMIS^®^ Dyspnea Characteristics v1.0 instrument (Table 4).

**Table 4:** Internal consistency reliability of the PROMIS^®^ instruments

PROMIS^®^ Instrument	No. of Items	Cronbach’s Alpha	ω_t_	Interpretation
Dyspnea Characteristics v1.0	5	0.896	0.911	Good-Excellent
Dyspnea Functional Limitations 10a	10	0.909	0.912	Excellent
Global Health v1.2	10	0.846	0.852	Good
Global Physical Health	4	0.649	0.664	Questionable
Global Mental Health	4	0.759	0.767	Acceptable

While the PROMIS^®^ Dyspnea Characteristics v1.0 instrument highlights multiple facets of dyspnea (e.g., occurrence, intensity, frequency, and duration), prior literature supports its use as a single summary measure of dyspnea severity [18]. As such, we modeled this instrument as a unidimensional reflective construct and computed a raw composite dyspnea severity score. CFA was estimated using DWLS, which is highly appropriate for ordinal data, and it allows for mixed response formats by modeling polychoric correlations [36]. Our analysis supports the use of this instrument as a single latent construct in this population.

### PROMIS^®^ Dyspnea Functional Limitations 10a

Item-level statistics were examined to assess each item’s contribution to the overall scale reliability. Corrected item-total correlations ranged from 0.65 to 0.79, demonstrating strong positive associations between each item and the overall score. This suggests good homogeneity among the items and that they collectively measure the same underlying construct. Additionally, the “Cronbach’s alpha if item deleted” values were all lower than the overall scale alpha, indicating that removal of any single item would not improve, and would likely reduce, the internal consistency of the instrument. Both values (α = 0.909; ω_t_ = 0.912) suggest excellent internal consistency and reliability within this instrument (Table 4). Item-level frequencies of “did not do” participant responses ranged from 0.242% to 36.232% across items (Appendix A1.2), with higher rates being observed for more physically demanding activities.

### PROMIS^®^ Global Health v1.2

Item-level statistics were examined to assess each item’s contribution to overall reliability for GPH and GMH. Corrected item-total correlations ranged from 0.34 to 0.74, indicating that each item demonstrated a moderate to strong positive association with the total score when the item itself was excluded. This suggests good homogeneity among the items and that they collectively measure the same underlying construct. Aside from Global10 (Emotional Problems), the “Cronbach’s alpha if item deleted” values were all lower than or equal to the overall scale alpha, indicating that the removal of any item would not improve, and would likely reduce, the internal consistency of the instrument. Both values (α = 0.846; ω_t_ = 0.852) suggest good internal consistency (Table 4). Additional analyses of the summary scores demonstrated differing reliability patterns (Table 4). The GMH showed acceptable internal consistency (α = 0.759; ω_t_ = 0.767). However, the GPH showed weaker internal consistency in our dysautonomia cohort (α = 0.649; ω_t_ = 0.664). While the PROMIS^®^ Global Health v1.2 instrument performed well, these findings suggest that the physical health summary score may be less internally homogeneous in this population.

### Construct validity

Confirmatory factor analysis was conducted to evaluate the underlying factor structure and overall model fit of each PROMIS^®^ instrument in this cohort.

### PROMIS^®^ Dyspnea Characteristics v1.0

This instrument provides a descriptive overview of an individual’s perception of dyspnea and was employed here as a single latent construct with a total score. This instrument demonstrated excellent model fit. All item standardized factor loadings were strong and significant on the latent factor (0.761–0.904; all *p* < 0.001), confirming a unidimensional factor structure (Table 5). The chi-square test was non-significant (χ² (5) = 6.34, *p* = 0.275), indicating no significant discrepancy between the observed and model-implied covariance matrices. The fit indices (CFI and TLI) were both 1.00, exceeding the conventional thresholds for a good fit and further supporting the model. The RMSEA was 0.025, and the test for close fit was non-significant (*p* = 0.742). The SRMR was 0.016, reflecting minimal residuals (Table 7). These results indicate that each item substantially contributed to measuring the underlying construct. As with previous research, these statistics provide sufficient evidence to support the use of a single-factor model [18].

**Table 5:** One-factor loadings for PROMIS^®^ Dyspnea Characteristics v1.0 (standardized regression coefficients)

Item	Standardized Loading
General Shortness of Breath	0.811
Occurrence of Shortness of Breath	0.904
Intensity of Shortness of Breath	0.840
Frequency of Shortness of Breath	0.876
Duration of Shortness of Breath	0.761

### PROMIS^®^ Dyspnea Functional Limitations 10a

This instrument was modeled as a unidimensional latent construct, which is consistent with its original validation and intended measurement framework [18]. CFA was estimated using DWLS, which is appropriate for ordinal response data. A single-factor model with zero added residual correlations provided an acceptable representation of the data (CFI = 0.974, TLI = 0.967), though initial RMSEA and SRMR values (0.209 and 0.125, respectively) were higher than acceptable standards, indicating a modest misfit. Examination of modification indices identified potential local dependencies between two conceptually related item pairs.

After reviewing the item content, we introduced two theoretically justified residual correlations: between “Lifting something 10–20 pounds” and “Moving something 10–20 pounds” (*r* = 0.663) and “Preparing meals” and “Washing dishes” (*r* = 0.552). These item pairs represent closely related physical activities and may share residual variance beyond the latent construct.

The revised model substantially improved the model fit (χ² (33) = 74.559, *p* < 0.001, CFI = 0.996, TLI = 0.994, RMSEA = 0.083, SRMR = 0.069) (Table 7). Although RMSEA remained slightly above the conventional 0.08 threshold, the remaining fit indices demonstrated very good model fit.

In the final model, all item standardized factor loadings were statistically significant (0.603–0.859; all *p* < 0.001) (Table 6). This supports a cohesive unidimensional structure. Together, these findings support the use of this instrument as a single-factor measure of dyspnea-related functional limitations in dysautonomia patients.

**Table 6:** One-factor loadings for PROMIS^®^ Functional Limitations 10a (standardized regression coefficients)

Item	Standardized Loading
Dressing yourself without help	0.738
Walking 50 steps without stopping	0.686
Walking up 20 stairs without stopping	0.767
Preparing meals	0.625
Washing dishes	0.603
Sweeping or mopping	0.827
Making a bed	0.859
Lifting something 10–20 pounds	0.822
Moving something 10–20 pounds	0.805
Walking about 0.5 miles without stopping	0.824

### PROMIS^®^ Global Health v1.2

We analyzed the PROMIS^®^ Global Health v1.2 instrument using CFA, which consisted of two correlated latent factors, GPH and GMH, each of which was defined by four distinct items [19]. Consistent with PROMIS^®^ scoring guidelines, Global01 (Global General Health) and Global09 (Global Social Health) were not included in the latent factor model; however, they were treated as standalone descriptive items [19, 28, 29].

Initially, all item standardized factor loadings were statistically significant for GPH (0.434–0.841; all *p* < 0.001) and GMH (0.561–0.886; all *p* < 0.001). The correlation between these two summary components was strong (*r* = 0.790, *p* < 0.001), which indicates a substantial positive and non-redundant relationship between the domains. The model fit indices exhibited a mixed fit with acceptable incremental fit (CFI = 0.948, TLI = 0.924) with poor absolute fit as the RMSEA and SRMR were both above the traditionally accepted values (0.151 and 0.099, respectively), suggesting possible model misfit and residual dependencies among items.

Following previous validation research, [19] we examined the modification indices and introduced residual correlations between related items. Specifically, we added residual correlations between Global04 (Mental Health) and Global10 (Emotional Problems). The resulting model fit was substantially improved (χ² (18) = 77.527, *p* < 0.001, CFI = 0.983, TLI = 0.973, RMSEA = 0.090, SRMR = 0.068) (Table 7). Although RMSEA remained slightly above the conventional threshold, the remaining fit indices supported good overall fit.

In the final model, all item standardized factor loadings were statistically significant for GPH (0.434–0.833; all *p* < 0.001) and GMH (0.329–0.929; all *p* < 0.001) (Table 8). There was an increase in the correlation between the two factors (*r* = 0.822, *p* < 0.001), which suggests that there is a strong but distinct relationship between physical and mental health constructs in this patient population.

**Table 7:** Summary of CFA results

PROMIS^®^ Instrument	χ² (df)	CFI	TLI	RMSEA	SRMR	Fit Interpretation
Dyspnea Characteristics v1.0	6.34 (5)	1.00	1.00	0.025	0.016	Excellent
Functional Limitations 10a	74.559 (33)	0.996	0.994	0.083	0.069	Very good
Global Health v1.2 (Two-Factor)	77.527 (18)	0.983	0.973	0.090	0.068	Good

**Table 8:** Two-factor loadings for PROMIS^®^ Global Health v1.2 (standardized regression coefficients)

Global Physical Health	Standardized Loading	
Global03 (Physical Health)	0.833	
Global06 (Physical Function)	0.695	
Global07 (Pain)	0.434	
Global08 (Fatigue)	0.517	
**Global Mental Health**	**Standardized Loading**	
Global02 (Quality of Life)	0.929	
Global04 (Mental Health)	0.625	
Global05 (Social Discretionary)	0.691	
Global10 (Emotional Problems)	0.329	

Global01 (Global General Health) and Global09 (Global Social Health) were descriptively examined and included in concurrent validity analyses [19, 28, 29]. These items showed weak correlations with the severity of dyspnea, which indicates that they capture broader aspects of overall health and social functioning above and beyond dyspnea-specific limitations.

It is important to note that Global10 (Emotional Problems) had a factor loading of 0.329. Before adding the correlation, the factor loading was 0.560, which is acceptable. The decrease in factor loading after adding a correlated residual between Global04 (Mental Health) and Global10 (Emotional Problems) suggests that some variance previously attributed to the latent factor is better explained by shared item-specific covariance. Additionally, this may be explained by the overlap of emotional and somatic symptoms (e.g., palpitations, cognitive dysfunction, fatigue, and shakiness) in people affected by autonomic nervous system dysfunction. Alternatively, emotional distress in people affected by chronic diseases like dysautonomia can be exacerbated by disease processes (e.g., diagnostic delay and functional impairment) [20].

### Concurrent criterion-related validity

Concurrent criterion-related validity was evaluated by correlating PROMIS^®^ Dyspnea Characteristics v1.0 raw composite scores with PROMIS^®^ Functional Limitations 10a T-scores and PROMIS^®^ Global Health v1.2 T-scores and descriptive single-item domains (Table 9). A strong positive correlation was observed between PROMIS^®^ Dyspnea Characteristics v1.0 and PROMIS^®^ Dyspnea Functional Limitations 10a (*r* = 0.59063), indicating that higher PROMIS^®^ Dyspnea Characteristics v1.0 raw scores, reflecting greater dyspnea severity, were associated with greater functional impairment. In contrast, correlations between PROMIS^®^ Dyspnea Characteristics v1.0 and PROMIS^®^ Global Health v1.2 summary scores (GMH and GPH) and single-item domains (Global Social Health and Global General Health) were weak to moderate (*r* = -0.17763 to -0.35034), reflecting the broader, multidimensional nature of general health compared with specific dyspnea-related limitations.

**Table 9:** Concurrent criterion-related validity of PROMIS^®^ instruments

PROMIS^®^ Instrument	Pearson’s *r*	Correlation Interpretation
Dyspnea Functional Limitations 10a	0.59063	Strong
Global Health v1.2: General Health	-0.19103	Weak
Global Health v1.2: Mental Health	-0.21698	Weak
Global Health v1.2: Social Health	-0.17763	Weak
Global Health v1.2: Physical Health	-0.35034	Moderate

## Discussion

The aim of this study was to validate three PROMIS^®^ instruments, Dyspnea Characteristics v1.0, Dyspnea Functional Limitations 10a, and Global Health v1.2, in a cohort of adults with dysautonomia. Although these instruments are validated in the general and in a few specific patient populations, [38–40] their performance in individuals with ANS disorders had not previously been examined. Given the high prevalence of dyspnea in dysautonomia [2, 4] and the absence of standardized tools validated for this patient population, we aimed to determine whether these PROMIS^®^ instruments reliably measure dyspnea severity, functional limitations, and quality of life in people with autonomic dysfunction. Our findings demonstrate that all three PROMIS^®^ instruments exhibit overall good to excellent reliability and good to excellent construct validity in this population, supporting their use in both clinical and research settings.

### Reliability and item performance

All three instruments exhibited sufficient internal consistency for psychometric adequacy in this patient population. The PROMIS^®^ Dyspnea Characteristics v1.0 and PROMIS^®^ Dyspnea Functional Limitations 10a instruments demonstrated good to excellent internal consistency. The PROMIS^®^ Global Health v1.2 instrument demonstrated good reliability for the overall instrument, while the reliability of its summary scores varied. GMH demonstrated acceptable internal consistency, whereas GPH showed weaker internal consistency in this dysautonomia cohort. A notable observation was the relatively low loading of Global10 (Emotional Problems) on the Global Mental Health instrument. This may be due to the shared variance with Global04 (Mental Health), or the overlap of orthostatic and emotional symptoms (e.g., palpitations, dizziness, tremulousness, cognitive dysfunction, and fatigue) in this patient population [43]. Moreover, chronic illness–related stressors, including diagnostic delay, symptom unpredictability, and functional limitations, may further complicate the relationship between emotional burden and autonomic symptoms [20]. Despite this lower factor loading, the GMH demonstrated acceptable reliability.

### Construct validity

Construct validity for all instruments was assessed confirmatory factor analysis (CFA), which supported unidimensional structures for the PROMIS^®^ Dyspnea Characteristics v1.0 and PROMIS^®^ Dyspnea Functional Limitations 10a instruments, consistent with their intended design and validation in the general population [7]. High factor loadings across items indicated that each survey instrument measured a single cohesive construct. While the initial fit indices for the PROMIS^®^ Dyspnea Functional Limitations 10a indicated a potential modest misfit, the model fit improved after including two theoretically justified residual correlations between two closely related physical tasks (i.e., “Lifting something 10–20 pounds” and “Moving something 10–20 pounds” as well as “Preparing meals” and “Washing dishes”). Three reasons may explain this misfit. First, RMSEA is sensitive to model complexity and degrees of freedom. In models with small degrees of freedom, RMSEA can be inflated when other fit indices indicate good fit. Second, the nature of the ordinal data and potential local dependencies among items could contribute to elevated residual-based fit indices. Finally, there was a moderate number of missing entries (approximately 14.9%), which could result in inflated RMSEA and SRMR values. Given the strong factor loadings and improved model fit, the evidence supports the use of a single-factor model.

For the PROMIS^®^ Global Health v1.2 instrument, the two-factor structure, which represents GPH and GMH, demonstrated a good model fit after we incorporated the theoretically justified correlation [19]. Consistent with the established PROMIS^®^ scoring methods, we analyzed the standard 8-item structure, with four items loading on each latent construct using CFA [19]. We did not include Global01 (Global General Health) and Global09 (Global Social Health) in the latent factor model and instead treated them only as descriptive indicators [28, 29].

The GPH and GMH factors showed a strong correlation, which suggests a substantial interdependence between physical and mental health in individuals with dysautonomia. This is consistent with the understanding that autonomic nervous system dysfunction affects multiple physiological and psychosocial domains, such as fatigue, pain, exercise tolerance, anxiety, and depression [43].

Descriptively, Global01 (Global General Health) and Global09 (Global Social Health) demonstrated a weaker association with dyspnea severity compared with the overall GPH summary score. This suggests that overall perceived health and social role functioning reflect broader dimensions of health that go beyond dyspnea-specific symptom burden. Together, these findings support using the PROMIS^®^ Global Health v1.2 instrument as a multidimensional measure of overall health that captures both domain-specific as well as global perceptions of well-being in this patient population.

### Concurrent validity and clinical implications

The strong correlation between PROMIS^®^ Dyspnea Characteristics v1.0 and PROMIS^®^ Dyspnea Functional Limitations 10a indicates that self-reported severity of dyspnea is closely linked with functional limitations in daily activities. This is consistent with clinical studies reporting that dysautonomia patients experience dyspnea during light or routine tasks or with orthostasis [7, 44, 45], likely due to a combination of neurologically-mediated cardiopulmonary mechanisms and hypovolemia [5, 12, 41, 42]. In contrast, correlations between the PROMIS^®^ Dyspnea Characteristics v1.0 raw scores, which we used to classify dyspnea severity, and PROMIS^®^ Global Health v1.2 were weak to moderate, suggesting that dyspnea represents only a part of the total symptom burden affecting people with dysautonomia. The moderate association with GPH suggests that participants’ ability to function physically is at least in part impacted by dyspnea. Together, these analyses indicate that PROMIS^®^ Dyspnea Functional Limitations 10a and PROMIS^®^ Global Health v1.2 measure distinct but complementary components of the participants’ health. Thus, these tools can be used in clinical and research settings to assess the impact of dyspnea on the patient’s ability to conduct daily activities and to evaluate the extent to which this symptom affects different domains of the patient’s quality of life.

### Limitations

As this study relied on patient-reported symptoms and diagnoses, it is subject to several limitations. First, although we asked participants to exclusively identify their formal diagnoses from pre-populated lists, participants may inadvertently or incorrectly report their conditions in the Dysautonomia and Comorbid Conditions instrument. To reduce ambiguity, we offered detailed lists of diagnoses and asked that participants only identify formal diagnoses made by their clinicians, and to indicate whether the diagnosis was in progress. Moreover, since different clinicians made the diagnoses of autonomic disorder and comorbid conditions under potentially different criteria (i.e., heterogeneity in clinical diagnostic practices), any additional statistical analyses to characterize the effect of specific forms of dysautonomia on dyspnea were not possible. Moreover, previous work has highlighted a potential mismatch in reported symptom burden and clinical autonomic testing, suggesting that additional studies correlating patient-reported symptoms and clinical testing are warranted [46]. Finally, as with any survey study, social desirability bias can affect participant responses, albeit perhaps less so in self-administered instruments [47].

Moreover, missing data in the PROMIS^®^ Dyspnea Functional Limitations 10a instrument might not be missing at random. Individuals with greater dyspnea severity might be less likely to attempt certain activities, especially if they are physically demanding or challenging. Although pairwise deletion was used in the primary analysis and sensitivity analyses demonstrated robustness of the results, this approach might introduce bias in both covariance estimates and model parameters under MNAR conditions.

## Conclusions

The aim of this study was to validate three PROMIS^®^ instruments in a dysautonomia cohort: Dyspnea Characteristics v1.0, Dyspnea Functional Limitations 10a, and Global Health v1.2. Statistical analyses indicate good to excellent internal consistency for PROMIS^®^ Dyspnea Characteristics v1.0 and PROMIS^®^ Dyspnea Functional Limitations 10a, as well as good overall internal consistency for PROMIS^®^ Global Health v1.2, although reliability varied between the GPH and GMH summary scores. Thus, these instruments can be used in clinical and research settings to assess the effects of dyspnea on the ability to conduct daily activities and on quality of life in people with dysautonomia.

## Data Availability

All data supporting the findings of this study are available within the paper and its Appendices. Instrument raw data and T-scores are available upon request.

## Appendix

**Table A1.1:** PROMIS^®^ dyspnea functional limitations instrument 10a

Considering your shortness of breath over the past 7 days, rate the amount of difficulty you had when doing the following activities:		
Item #	Functional Task	Response Options with Assigned Numerical Value Based on Difficulty of Execution*
**1**	Dressing yourself without help	1 = No difficulty 2 = A little difficulty 3 = Some difficulty 4 = Much difficulty
**2**	Walking 50 steps/paces on flat ground at a normal speed without stopping	1 = No difficulty 2 = A little difficulty 3 = Some difficulty 4 = Much difficulty
**3**	Walking up 20 stairs (2 flights) without stopping	1 = No difficulty 2 = A little difficulty 3 = Some difficulty 4 = Much difficulty
**4**	Preparing meals	1 = No difficulty 2 = A little difficulty 3 = Some difficulty 4 = Much difficulty
**5**	Washing dishes	1 = No difficulty 2 = A little difficulty 3 = Some difficulty 4 = Much difficulty
**6**	Sweeping or mopping	1 = No difficulty 2 = A little difficulty 3 = Some difficulty 4 = Much difficulty
**7**	Making a bed	1 = No difficulty 2 = A little difficulty 3 = Some difficulty 4 = Much difficulty
**8**	Lifting something weighing 10–20 lbs (about 4.5–9 kg, like a large bag of groceries)	1 = No difficulty 2 = A little difficulty 3 = Some difficulty 4 = Much difficulty
**9**	Carrying something weighing 10–20 lbs (about 4.5–9 kg, like a large bag of groceries) from one room to another	1 = No difficulty 2 = A little difficulty 3 = Some difficulty 4 = Much difficulty
**10**	Walking (faster than your usual speed) for ½ mile (almost 1 km) without stopping	1 = No difficulty 2 = A little difficulty 3 = Some difficulty 4 = Much difficulty

*The instrument was administered to participants as it was originally validated [18]; therefore, numerical values were not visible to respondents. Individuals who answered, “I did not do this activity in the last 7 days” were assigned a blank score for that question (Table A.1.2)

**Table A1.2:** Item-level frequency of “Did Not Do” responses for PROMIS^®^ Dyspnea Functional Limitations 10a

Functional Task	Frequency (*n*)	Prevalence (%)
Dressing yourself without help	1	0.242
Walking 50 steps/paces on flat ground at a normal speed without stopping	26	6.280
Walking up 20 stairs (2 flights) without stopping	84	20.290
Preparing meals	28	6.763
Washing dishes	56	13.527
Sweeping or mopping	105	25.362
Making a bed	73	17.633
Lifting something weighing 10–20 lbs (about 4.5–9 kg, like a large bag of groceries)	52	12.560
Carrying something weighing 10–20 lbs (about 4.5–9 kg, like a large bag of groceries) from one room to another	72	17.391
Walking (faster than your usual speed) for ½ mile (almost 1 km) without stopping	150	36.232

**Table A2.1:** Dysautonomia and Comorbid Conditions instrument

Item #	Dysautonomia Diagnosis	
	Clinical History	Response Options
**1**	Do you have a formal diagnosis made by a physician?	• Yes • No
**2**	Year the diagnosis was made (If you do not remember, please type: 0000)	• Entered response
**3**	Year of symptom onset (example: 2010) (If you do not remember, please type: 0000)	• Entered response
**4**	What type of clinical specialist made your diagnosis?	• Neurologist • Cardiologist (includes electrophysiologist) • Pulmonologist • Internal medicine doctor • Family medicine doctor • Rheumatologist • Physical medicine and rehabilitation (PM and R) doctor • Other
**5**	Other clinician who made dysautonomia diagnosis (Please do not indicate personal name)	• Entered response
**6**	Was the diagnosis made at a large academic medical center?	• Yes • No • I am not sure
**7**	Please indicate the type of dysautonomia you have. If you are not sure, or if evaluation is in progress, please select “I don’t know.”	• Postural orthostatic tachycardia syndrome (POTS) • Neurocardiogenic syncope (NCS), vasovagal Syncope (VVS), neurally mediated syncope (NMS) • Multiple system atrophy (MSA) • Pure autonomic failure (PAF) • Orthostatic hypotension (OH) • Inappropriate sinus tachycardia (IST) • Autoimmune autonomic ganglionopathy (AAG) • Baroreflex failure (BF) • Familial dysautonomia (FD) • Rheumatic autoimmune-related dysautonomia (such as Sjogren’s syndrome, rheumatoid arthritis, lupus, ankylosing spondylitis, and systemic sclerosis/scleroderma) • Other autoimmune-mediated dysautonomia • I don’t know • Long COVID-related dysautonomia • Post concussive dysautonomia (i.e., following mild traumatic brain injury) • Other
**8**	Type name of “Other form of dysautonomia”.	• Entered response
**9**	Did dysautonomia develop after a head injury?	• Yes • No
	**Co-Occurring Conditions**	**The following questions will help us identify whether you have been formally diagnosed with other medical conditions that are common in the dysautonomia patient population.**
**Item #**	**Clinical History**	**Response Options**
**1**	Please indicate whether you have a formal diagnosis of any of these conditions that can co-occur in people with dysautonomia.	• Mast cell disorders (including mast cell activation syndrome (MCAS), mast cell activation disorder (MCAD), and mastocytosis) • Ehler-Danlos syndrome (EDS) • Chiari malformation • Sjogren’s syndrome • Systemic lupus erythematosus (SLE) • Hashimoto’s thyroiditis • Antiphospholipid syndrome (APS) • Sarcoidosis • Celiac disease • Ulcerative colitis • Fibromyalgia • Myalgic encephalomyelitis (ME) / chronic fatigue syndrome (CFS) • Parkinson’s disease • Amyloidosis • Diabetes • Chronic inflammatory demyelinating polyneuropathy (CIDP) • Delta storage pool deficiency • Mitochondrial disease • Charcot-Marie-Tooth (CMT) disease • Paraneoplastic syndrome • Vitamin deficiency (includes vitamin E, B1, B3, B6, B12) • None of these conditions apply to me • Long COVID • Traumatic brain injury (TBI), including concussion • Other
**2**	Type the name of the condition (other than dysautonomia).	• Entered response
	**Conditions That May Cause Shortness of Breath**	**The following questions will help us to identify whether you have other conditions that may also cause shortness of breath.**
**Item #**	**Clinical History**	**Response Options**
**1**	Please indicate whether you have a formal diagnosis of any of these conditions that may also cause shortness of breath.	• Severe anemia • Obesity • Interstitial lung disease (ILD) (any type of pulmonary fibrosis) • Chronic obstructive pulmonary disease (COPD), emphysema, chronic bronchitis • Asthma • Pulmonary hypertension • Lung cancer • Pulmonary embolism • Untreated obstructive sleep apnea (OSA) • Other lung disease • Neuromuscular disease • Heart valvular disease • Heart failure • Cardiomyopathy • History of heart attack • Coronary artery disease (CAD) • Other heart disease • Mitochondrial disease • None of these applies to me • Other conditions that may cause shortness of breath
**2**	If you selected “Other Lung Disease,” please indicate the name of the lung condition	• Entered response
**3**	If you selected “Other heart disease,” please indicate the type	• Entered response
**4**	If you selected “Other” disease causing shortness of breath, please indicate the name of the disease	• Entered response
	**COVID-19**	**Since COVID-19 can cause lung damage and dysfunction of the autonomic nervous system**,** it’s important that we include some information about this disease in our analysis.**
**Item #**	**COVID-19 History**	**Response Options**
**1**	Have you had COVID-19?	• Yes • No
**2**	Did you develop dysautonomia as a result of COVID-19 infection (i.e., post-viral syndrome)?	• Yes • No
**3**	If you already had dysautonomia, have your dysautonomia symptoms worsened after COVID-19?	• Yes • No
**4**	Have you been diagnosed with Long Covid? Please note that Long COVID has many names, including post-COVID conditions, long-haul COVID, post-acute COVID-19, long-term effects of COVID, and chronic COVID, and post-acute sequelae of SARS-CoV-2 infection (PASC).	• Yes • No
**5**	Have you been vaccinated for COVID-19?	• Yes • No

**Table A3.1:** PROMIS^®^ Dyspnea Functional Limitations 10a fit indices

Fit Index	Original Two-Residual	Sensitivity Analysis Two-Residual
χ² (df)	74.559 (33)	130.025 (33)
p-value	< 0.001	< 0.001
CFI	0.996	0.993
TLI	0.994	0.991
RMSEA	0.083	0.085
SRMR	0.069	0.057

**Table A3.2:** PROMIS^®^ Dyspnea Functional Limitations 10a factor loadings

Item	Original Two-Residual	Sensitivity Analysis Two-Residual
Dressing yourself	0.738	0.679
Walking 50 steps	0.686	0.754
Walking up 20 stairs	0.767	0.720
Preparing meals	0.625	0.744
Washing dishes	0.603	0.707
Sweeping/mopping	0.827	0.785
Making a bed	0.859	0.762
Lifting 10–20 lbs	0.822	0.685
Moving 10–20 lbs	0.805	0.699
Walking 0.5 miles	0.824	0.716

**Table A3.3:** PROMIS^®^ Dyspnea Functional Limitations 10a reliability indices

Model	Original Two-Residual	Sensitivity Analysis Two-Residual
Cronbach’s α	0.909	0.898
McDonald’s ω	0.912	0.899

## Abbreviations

ANS: Autonomic Nervous System
POTS: Postural Orthostatic Tachycardia Syndrome
PROMIS: Patient-Reported Outcomes Measurement Information System
CFA: Confirmatory Factor Analysis
DWLS: Diagonally Weighted Least Squares
CFI: Comparative Fit Index
TLI: Tucker-Lewis Index
RMSEA: Root Mean Square Error of Approximation
SRMR: Standardized Root Mean Square Residual
